# Influence of drainage and nutrient-solution nitrogen and potassium concentrations on the agronomic behavior of bell-pepper plants cultivated in a substrate

**DOI:** 10.1371/journal.pone.0180529

**Published:** 2017-07-05

**Authors:** Anderson Fernando Wamser, Arthur Bernardes Cecilio Filho, Rodrigo Hiyoshi Dalmazzo Nowaki, Juan Waldir Mendoza-Cortez, Miguel Urrestarazu

**Affiliations:** 1 Estação Experimental de Caçador, Empresa de Pesquisa Agropecuária e Extensão Rural de Santa Catarina, Caçador, Santa Catarina, Brazil; 2 Departamento de Produção Vegetal, Faculdade de Ciências Agrárias e Veterinárias, Universidade Estadual Paulista, Jaboticabal, São Paulo, Brazil; 3 Departamento de Fitotecnia, Facultad de Agronomía, Universidad Nacional Agraria La Molina, Lima, Peru; 4 Departamento de Agronomía, Escuela Superior de Ingeniería, Universidad de Almería, Almería, Almería, Spain; University of Delhi, INDIA

## Abstract

The interactive effects of N (6, 9, 12 and 15 mmol L^-1^) and K (3, 5, 7, and 9 mmol L^-1^) concentrations in nutrient solutions were evaluated on bell pepper grown in a coconut-coir substrate and fertilized without drainage. An additional treatment with drainage was evaluated using N and K concentrations of 12 and 7 mmol L^-1^, respectively. The hybrid Eppo cultivar of yellow bell pepper was cultivated for 252 days beginning 9 November 2012. Electrical conductivity (EC), the N and K concentrations in the substrate solution, marketable fruit yield, total dry weight and macronutrient concentrations in shoots were periodically evaluated. Fruit production was lower in the system without drainage, regardless of the N and K concentrations, compared to the recommended 10–20% drainage of the volume of nutrient solution applied. Higher K concentrations in the nutrient solution did not affect plant production in the system without drainage for the substrate with an initial K concentration of 331.3 mg L^-1^. Fruit yield was higher without drainage at a nutrient-solution N concentration of 10.7 mmol L^-1^. The upper EC limit of the substrate solution in the system without drainage was exceeded 181 days after planting. Either lower nutrient concentrations in the nutrient solution or a drainage system could thus control the EC in the substrate solution.

## Introduction

The management of plant nutrition in substrate cultivation is based on the application of a complete nutrient solution by fertigation at a specific percentage of drainage of the volume applied [1]. Draining renews the substrate solution and prevents its salinization by leaching excess nutrients and other elements not absorbed by plants between each fertigation [2]. The drained nutrient solution can be stored in the reservoir to be reused in a closed system or can be directly discarded into the soil in an open or free-drainage system. This latter system is more common in substrate cultivation in Brazil. Soil-less cultivation with free drainage, however, has been associated with losses of large volumes of water and nutrients and with the contamination of groundwater from the release of nitrates [3, 4]. Efforts have thus recently been applied to develop management practices that reduce the release of nutrients by drainage [4–7].

The drainage percentage is a function of the quality of the irrigation water, specifically its electrical conductivity (EC). As the lower is the water EC, the lower is the drainage percentage [8]. Brazil has vast water resources and excellent water quality that can be used in agriculture, with low salinity (EC <0.25 dS m^-1^) and sodium content [9]. We propose a system of fertigation management for cultivation using a substrate in which the nutrient solution, using water of good quality, is applied by fertigation based on the evapotranspirative demand of the crop without excessive application of the nutrient solution and without its drainage. Studies evaluating the cultivation of melon [10] and tomato [11] in substrates without drainage have reported successful results with this system. The relatively short cycles evaluated for these cultures, however, may have limited the increase in substrate EC and thus damage to the crop. The validation of the proposed system (no drainage) is thus needed for crops with longer cycles, such as bell pepper, whose cycle in greenhouse conditions may extend for more than 10 months [12].

Fertigation without drainage may modify the nutrient requirement of plants due to nutrient accumulation in the substrate solution. Determining the concentrations of nutrients in the nutrient solution for promoting proper growth and production of the plants using this system is thus necessary. Bell pepper [13] requires high quantities of N and K, so determining their appropriate concentrations in the nutrient solution is of utmost importance. We tested the hypothesis that low N and K concentrations in the nutrient solution could be adequate in bell-pepper fertigation in an undrained system, when irrigation water with low EC are used. The main aim of this study was thus to determine the optimal N and K concentrations in the nutrient solution for bell-pepper cultivation fertigated in an undrained substrate.

## Materials and methods

An experiment was conducted at the Julio de Mesquita Filho campus of the State University of São Paulo (UNESP), Jaboticabal, São Paulo (21°14'S, 48°17'W; 549 m a.s.l.) in a greenhouse with a low-density polyethylene cover 150 μm thick and side and front closure with black polypropylene mesh providing 50% shade. The greenhouse was 48 m long, 12.8 m wide and 3.3 m high and was oriented east-west. A thermo-reflective mesh with 50% shading was installed to control the temperature inside the greenhouse.

The hybrid Eppo cultivar of bell pepper (*Capsicum annuum* L.) was cultivated for 252 days beginning 9 November 2012 (mid-spring to early winter). Plants were grown 125 cm apart in 240-L channels 480 cm long, 20 cm wide and 25 cm deep filled with coconut coir and oriented north-south in the soil at a slope of 0.5%. Golden Mix^®^ 98 coconut-coir substrate was used (Amafibra, Pará, Brazil), whose chemical properties, based on the aqueous-extraction method 1:1.5 (v:v) [14], were: pH, 5.4; EC, 1.0 dS m^-1^; and in mg L^-1^, 3.3 of NO_3_^−^-N, 48.1 of P, 40.2 of Cl, 88.8 of S, 5.2 of NH_4_^+^-N, 331.3 of K, 12.3 of Na, 8.0 of Ca, 4.5 of Mg, 1.0 of B, 0.04 of Cu, 0.3 of Fe, 0.3 of Mn and 0.3 of Zn. Seedlings were grown in 162-cell trays (31 mL cell^-1^) filled with coconut-shell powder and vermiculite and transplanted at the four-leaf stage at twelve plants per channel. Planting density was 2.0 plants m^-2^ (40 × 125 cm). The plants were maintained with four main stems arranged in a "V" by horizontal plastic polythene strips parallel to the cultivation channels.

The nutrient solutions were applied by drip irrigation using a tube with 24 self-compensating and anti-drain drippers per cultivation channel (two drippers plant^-1^) at a flow rate of 1.8 L h^-1^ per dripper. After the dripline was installed, the substrate was covered with double-sided plastic with the white side facing up.

The foliage was sprayed weekly with calcium chloride (6 g L^-1^) from the beginning of fruit development (31 days after planting (DAP)) to prevent the onset of blossom-end rot in the fruit. The fruit was harvested weekly from 76 DAP and continued until the end of the cycle.

Combinations of four N concentrations (6, 9, 12 and 15 mmol L^-1^) and four K concentrations (3, 5, 7 and 9 mmol L^-1^) in the nutrient solution were evaluated. N and K concentrations were higher for two treatments and lower for two treatments than those proposed by Castellane and Araujo [15], namely 11 and 6.3 mmol L^-1^ of N and K, respectively. These 16 treatments (nutrient solutions) were applied without drainage of the nutrient solution. An additional treatment with the drainage of 10–20% of the fertigated volume was evaluated using N and K concentrations of 12 and 7 mmol L^-1^, respectively. The experimental design was a complete randomized block in a 4 × 4 + 1 factorial scheme with three replicates. Each of these 51 plots had a total area of 6.0 m^2^, corresponding to a growing channel containing 12 plants. Each side of the blocks was bordered by a channel with plants.

The N and K concentrations in the nutrient solutions followed levels established for each treatment. The concentrations of the other nutrients for all treatments were those proposed by Castellane and Araujo [15], which for macronutrients in mmol L^-1^ were 1.3 of P, 2.8 of Ca, 1.2 of Mg and 1.2 of S and for micronutrients in mg L^-1^ were 0.3 of B, 0.05 of Cu, 3.7 of Fe, 0.4 of Mn, 0.05 of Mo and 0.3 of Zn. Phosphoric and nitric acids were used to maintain the nutrient solutions at pH 6.0 and provide some of the P and N macronutrients, respectively. The sources and amounts of the nutrients are shown in Table 1. The ECs of the nutrient solutions were determined after their preparation (Table 1). The water used to prepare the solutions had the following chemical properties: EC, 0.2 dS m^-1^; pH, 7.6 and in mg L^-1^, 0.2 of N-NO_3_, 1.6 of K^+^ 20.1 of Ca^2+^, 1.2 of Mg^2+^, 15.5 of Na^+^, 3.3 of Cl^-^ and 87.0 of HCO_3_^-1^.

**Table 1:** Sources and amounts of salts and acids in the nutrient solutions and average EC based on the N and K concentrations evaluated.

Nutrient source	Desired concentration of N (mmol L^-1^)															
	6				9				12^z^				15			
	Desired concentration of K (mmol L^-1^)															
	3	5	7	9	3	5	7	9	3	5	7^z^	9	3	5	7	9
	Amount added (mmol L^-1^)															
Ca(NO_3_)_2_	2.6	2.6	2.6	2.6	2.8	2.6	2.6	2.6	3	2.8	2.7	2.6	3	3	3	2.8
KNO_3_	0.7	0.7	0.7	0.7	3.0	3.7	3.7	3.7	2.6	4.6	6.6	6.7	3	5	7	9
NH_4_NO_3_	0	0	0	0	0	0	0	0	1.7	0.9	0	0	1.9	1.8	0.8	0
NH_4_H_2_PO_4_	0	0	0	0	0.4	0	0	0	0	0	0	0	1.3	0.4	0.4	0.4
KH_2_PO_4_	0.4	0.4	0.4	0.4	0	0.4	0.4	0.4	0.4	0.4	0.4	0.4	0	0	0	0
MgSO_4_	1.2	1.2	1.2	1.2	1.2	1.2	1.2	1.2	1.2	1.2	1.2	1.2	1.2	1.2	1.2	1.2
KCl	1.9	3.9	5.9	7.9	0	0.9	2.9	4.9	0	0	0	1.9	0	0	0	0
H_3_PO_4_	0.9	0.9	0.9	0.9	0.9	0.9	0.9	0.9	0.9	0.9	0.9	0.9	0	0.9	0.9	0.9
HNO_3_	0	0	0	0	0	0	0	0	0	0	0	0	0.9	0	0	0
EC (dS m^-1^)	1.3	1.6	1.8	2.0	1.4	1.5	1.8	2.0	1.6	1.7	1.8	2.0	1.7	1.8	2.0	2.1

Micronutrients used were (mg L^-1^) 1.36 ZnSO_4_, 0.22 CuSO_4_, 1.54 MgSO_4_, 0.09 (NH_4_)_6_Mo_7_O_24_, 1.76 H_3_BO_3_ and 56.92 Fe-EDDHMA.

^z^ N and K concentrations used in the treatment with drainage.

The nutrient solutions of each treatment were manually prepared weekly in separate 500-L tanks. The substrate moisture content to indicate the time of fertigation was instantly determined by Irrigás^®^ sensors [16] (model PRO 15 kPa, Hidrosense, São Paulo, Brazil) installed in the treatments containing 12 and 7 mmol L^-1^ of N and K, respectively, with and without drainage. The sensors were connected to an electronic irrigation controller (Model MRI-10/2, Hidrosense, Sao Paulo, Brazil) by low-density polyethylene microtubes 8 mm in diameter. The plants were fertigated when the substrate moisture tension reached -4 kPa using the time required to fertigate the plants to the moisture-retention capacity of the substrate without drainage. Enough time was added in the treatment with 10–20% drainage, as proposed for irrigation water with an EC of 0–0.5 dS m^-1^ [1].

Substrate samples were collected 61, 132, 181 and 252 DAP. At each sampling, 2 L of substrate were systematically removed between every two plant per plot, starting from the first plant in a channel. The substrate solution was obtained by an aqueous-extraction method at 1:1.5 (v:v) [14]. The extract was used to determine the total-N concentrations by distillation and titration, and the K concentrations by Inductively Coupled Plasma—Atomic Emission Spectrometry (model Vista MPX, Varian, Mulgrave, Australia). EC was measured by a digital conductivity meter (Model 330i, WTW, Weilheim, Germany).

Three plants per plot were sampled at the end of the harvest. The plants were washed with tap water and then deionized water, dried in a forced-air oven at 65°C to a constant mass and weighed. Two fruits per harvest per plot were weighed and were similarly processed (washing, drying and weighing). The amount of dry matter of the harvested fruits was subsequently used to estimate the weight of the remaining fruit at the end of the cycle. Macronutrient content was evaluated in the sampled plants to assess the total amount of macronutrient in the plants [17]. Accumulations were obtained by multiplying the concentrations of the nutrients with the plant masses.

Fruits with >70% yellow color were harvested weekly. Fruits were classified into marketable and unmarketable. Only fruits with blossom-end rot were considered unmarketable, and all others were classified as marketable. The number and individual weights of fresh fruit were determined for each class.

The variables of fruit yield were evaluated for all harvests, and the average accumulated values were expressed for every 60 days of harvest (early, intermediate and late harvests) and the end of the harvest. Data were submitted to an analysis of variance by an *F* test at 5% error probability using the fat2.ad.dbc procedure of the ExpDes.pt R package [18] based on the analysis of a factorial experiment with additional treatments described by Healy [19].

## Results

### Chemical properties of the substrate

**Fig 1:**
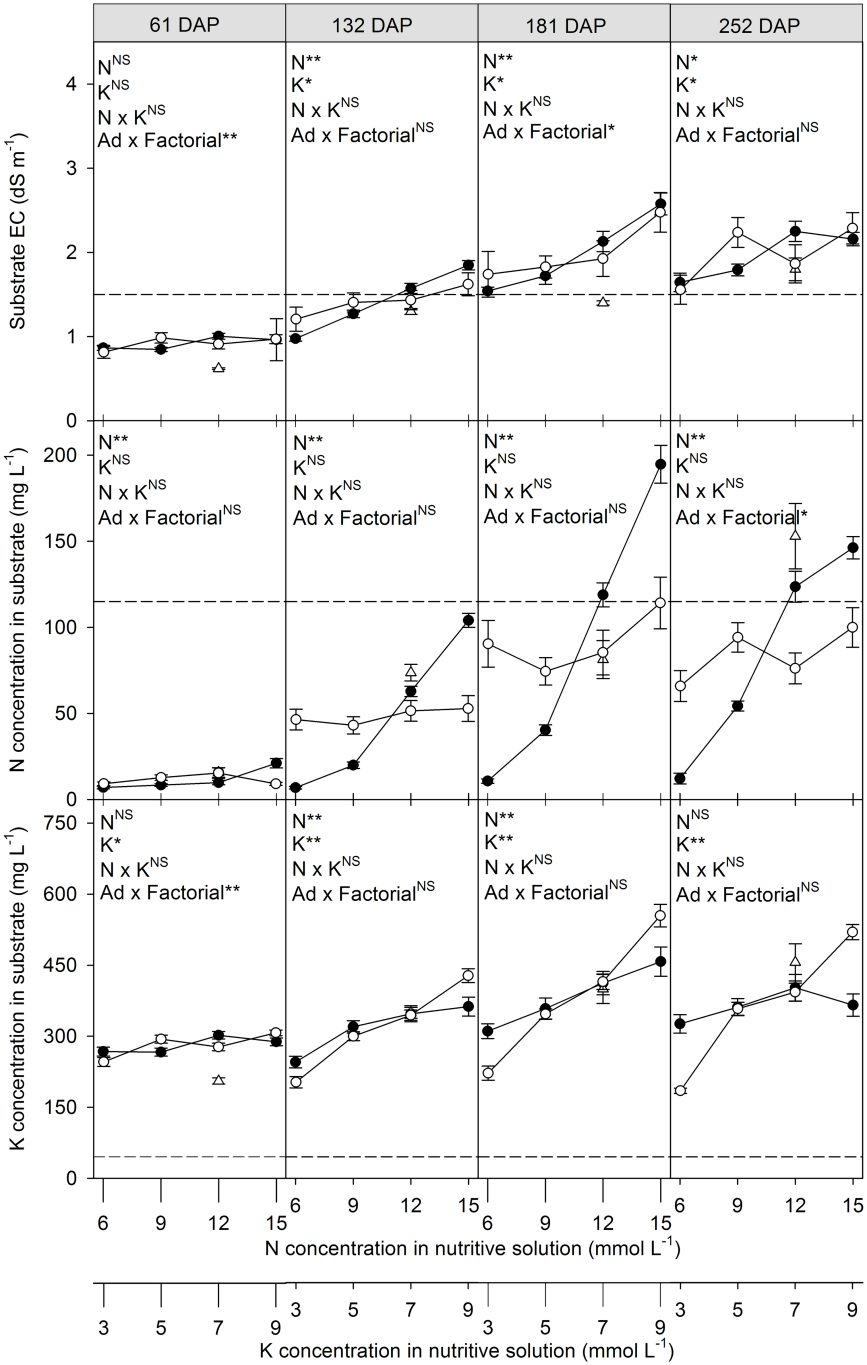
Evolution of electrical conductivity (EC), N concentration and K concentration in the substrate solution as a function of the nutrient-solution N (●) and K (○) concentrations for bell pepper grown in a fertigated substrate without drainage. The triangle (Δ) indicates the average additional treatment (Ad) with 10–20% drainage of the nutrient-solution volume. Vertical bars represent the standard error. N, K, N x K and Ad x Factorial represent, in the analysis of variance, the causes of variation factor "concentration of N", factor "concentration of K", interaction N versus K, additional treatment (with drainage) versus the factorial treatments (without drainage), respectively. Horizontal dashed lines represent the upper limits considered suitable for EC, N (NO_3_ + NH_4_) and K concentrations [20]. ^NS^, * and ** indicates F test not significant or significant at *p* = 0.05 or 0.01, respectively.

EC and the N and K concentrations of the substrate solution were not influenced by the interaction between the N and K concentrations (Fig 1 and S1 Table).

EC of the substrate solution increased over the cycle for all treatments without drainage and for the treatment with drainage (Fig 1 and S1 Table). EC also increased due to the increase in both the N and K concentrations in the nutrient solution from 132 DAP. EC was lower in the treatment with drainage than the treatments without drainage 61 and 181 DAP but not for the other evaluation periods. Concentrations of 12 and 15 mmol L^-1^ N and 9 mmol L^-1^ K 132 DAP exceeded the limit EC suitable for cultivation in substrate of 1.5 dS m^-1^ [20]. EC of all treatments without drainage exceeded this limit by 181 DAP. The treatment with drainage only exceeded the limit of 1.5 dS m^-1^ evaluated by 252 DAP.

The N concentration in the substrate was influenced only by the N concentration in the nutrient solution, and N concentrations increased in the substrate with increasing nutrient-solution N concentrations for all evaluation times (Fig 1 and S1 Table). The N concentration in the substrate also increased throughout the crop cycle, and the higher the N concentration in the nutrient solution, the greater the increases over the first period evaluated (61 DAP). It is noteworthy that the concentration of 6 mmol L^-1^ slightly altered the N concentration in the substrate solution over the evaluation times. N concentrations in the substrate increased by 45.9, 113.8 and 125.2 mg L^-1^ for the nutrient-solution N concentrations of 9, 12 and 15 mmol L^-1^, respectively, between the first (61 DAP) and last (252 DAP) evaluation times and by 5.1 mg L^-1^ for the nutrient-solution N concentration of 6 mmol L^-1^. The N concentrations in the substrate did not differ significantly between the treatments with and without drainage, except for the last evaluation time, when the N concentration was higher in the treatment with drainage. The limit N (NO_3_^-^ + NH_4_^+^) concentration considered appropriate in the substrate solution of 115 mg L^-1^ (80 mg L^-1^ NO_3_^-^ and 35 mg L^-1^ NH_4_^+^) [20] was exceeded in the last two evaluation times in the N concentrations of 12 and 15 mmol L^-1^ of the treatments without drainage, and the last evaluation times for the treatment with drainage.

The K concentrations in the substrate solution were influenced by the K concentration in the nutrient solution at all evaluation times and by the N concentration in the nutrient solution 132 and 181 DAP. The nutrient-solution K and N concentrations increased the substrate-solution K concentration. Nutrient-solution K concentrations of 5, 7 and 9 mmol L^-1^ increased the K concentration in the substrate throughout the crop cycle. It is noteworthy that the concentration of 3 mmol L^-1^ decreased the substrate-solution K concentration along the evaluation times. The substrate-solution K concentrations increased by 64.2, 115.8 and 212.6 mg L^-1^ for the nutrient-solution K concentrations of 5, 7 and 9 mmol L^-1^, respectively, between the first and last evaluation times and decreased by 61.1 mmol L^-1^ for the nutrient-solution K concentration of 3 mmol L^-1^. The K concentrations in the substrate did not differ significantly between the treatments with and without drainage, except for the first evaluation time (61 DAP) when the K concentration was lower in the treatment with drainage. The limit K concentration considered appropriate in the substrate solution of 45 mg L^-1^ [20], however, was exceeded in all treatments from the first evaluation time.

### Dry mass and accumulation of macronutrients in the plant shoots

The dry mass and accumulation of macronutrients in the plant shoots were not influenced by the interaction between the N and K concentrations (Table 2 and S2 Table). The nutrient-solution K concentration did not affect the amount of dry matter or the accumulation of macronutrients in the shoots of the bell-pepper plants. The nutrient-solution N concentrations higher than 9 mmol L^-1^ increased the dry matter and accumulation of N and K in the plant shoots, with no differences until the concentration of 15 mmol L^-1^. The dry mass and accumulation of macronutrients did not differ significantly between the treatments with and without drainage.

**Table 2:** Influence of the nutrient-solution N and K concentrations without drainage on the dry mass and macronutrients in the shoots.

Factor	Dry mass	Macronutrient accumulation				
		(g plant^-1^)				
	(g plant^-1^)	N	K	P	Ca	Mg
N concentration (mmol L^-1^)						
6	436.4	11.2	14.7	2.5	2.4	1.1
9	512.7	12.5	17.9	3.0	2.5	1.4
12	495.3	13.2	17.2	2.7	2.4	1.3
15	502.1	13.5	17.3	2.8	2.4	1.3
Statistical significance	LSD = 43.9	LSD = 0.8	LSD = 1.5	NS	NS	NS
K concentration (mmol L^-1^)						
3	482.4	13.0	16.4	2.8	2.4	1.3
5	484.7	12.3	16.9	2.7	2.5	1.3
7	471.8	12.1	16.0	2.5	2.3	1.2
9	507.8	13.0	17.7	2.9	2.5	1.3
Statistical significance	NS	NS	NS	NS	NS	NS
N *vs* K	NS	NS	NS	NS	NS	NS
With drainage^w^	568.8	13.8	20.9	3.2	2.7	1.6
With *vs* without drainage	NS	NS	NS	NS	NS	NS

LSD, least significant difference at *p* = 0.05.

NS, not significant at *p* = 0.05.

^w^ fertigation of nutrient solution using N and K concentrations of 12 and 7 mmol L^-1^, respectively, with 10–20% drainage of the volume applied.

### Production of marketable fruits

**Fig 2:**
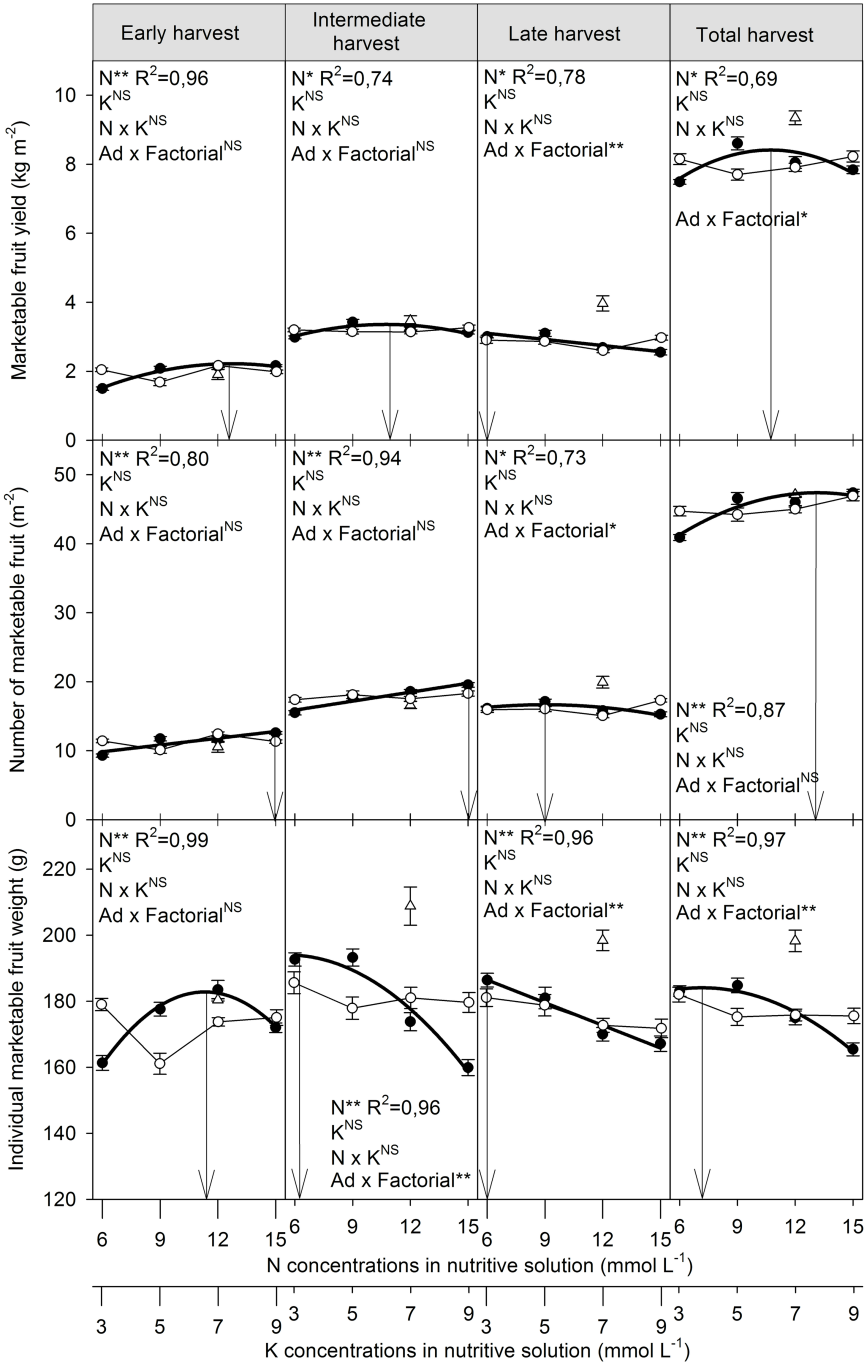
Yield and number and weight of marketable fruits per evaluation period and total as a function of the nutrient-solution N (●) and K (○) concentration for bell pepper grown in the substrate without drainage. The triangle (Δ) indicates the average additional treatment (Ad) with 10–20% drainage of the nutrient-solution volume. Vertical bars represent the standard error. N, K, N x K and Ad x Factorial represent, in the analysis of variance, the causes of variation factor "concentration of N", factor "concentration of K", interaction N versus K, additional treatment (with drainage) versus the factorial treatments (without drainage), respectively. Arrows indicate the N concentration for obtaining the maximum technical efficiency. ^NS^, * and ** indicates F test not significant or significant at *p* = 0.05 or 0.01, respectively. R^2^ indicates the coefficient of determination of the adjusted polynomial equations.

Yield and number and weight of marketable fruits were not affected by the K concentration in the nutrient solution, and these variables were not influenced by the interaction between the N and K concentrations (Fig 2 and S3 Table).

Higher N concentrations in the nutrient solution increased the marketable yield in all crops, with an estimated maximum yield of 8.4 kg m^-2^ at a concentration of 10.7 mmol L^-1^ (Fig 2 and S3 Table). Nutrient-solution N concentrations of 12.6, 10.9 and 6.0 mmol L^-1^ maximized estimated yields in the early, intermediate and late harvests, respectively. Yield was higher in the treatment with than without drainage for the late harvest period, as well as in the total harvest. Yield did not differ significantly between the treatments with and without drainage for the first two harvest periods.

The estimated number of marketable fruits per square meter was highest (47.4) at a nutrient-solution N concentration of 13.1 mmol L^-1^ (Fig 2 and S3 Table). The estimated marketable fruit weight was highest (184.1 g) at an N concentration of 7.2 mmol L^-1^ (Fig 2 and S3 Table). The estimated numbers of fruits in the early, intermediate and late harvests were highest at N concentrations of 15.0, 15.0 and 9.0 mmol L^-1^, respectively. The fruit weights in the early, intermediate and late harvests were highest at N concentrations of 11.4, 6.2 and 6.0 mmol L^-1^, respectively.

The number of fruits was higher in the treatment with than without drainage only in the last harvest period. The number of fruits, however, did not differ significantly between the treatments with and without drainage considering the total harvest. Fruit weight was higher in the treatment with drainage than in those without drainage in the intermediate and late harvests, as well as considering the total harvest.

### Blossom-end rot

**Fig 3:**
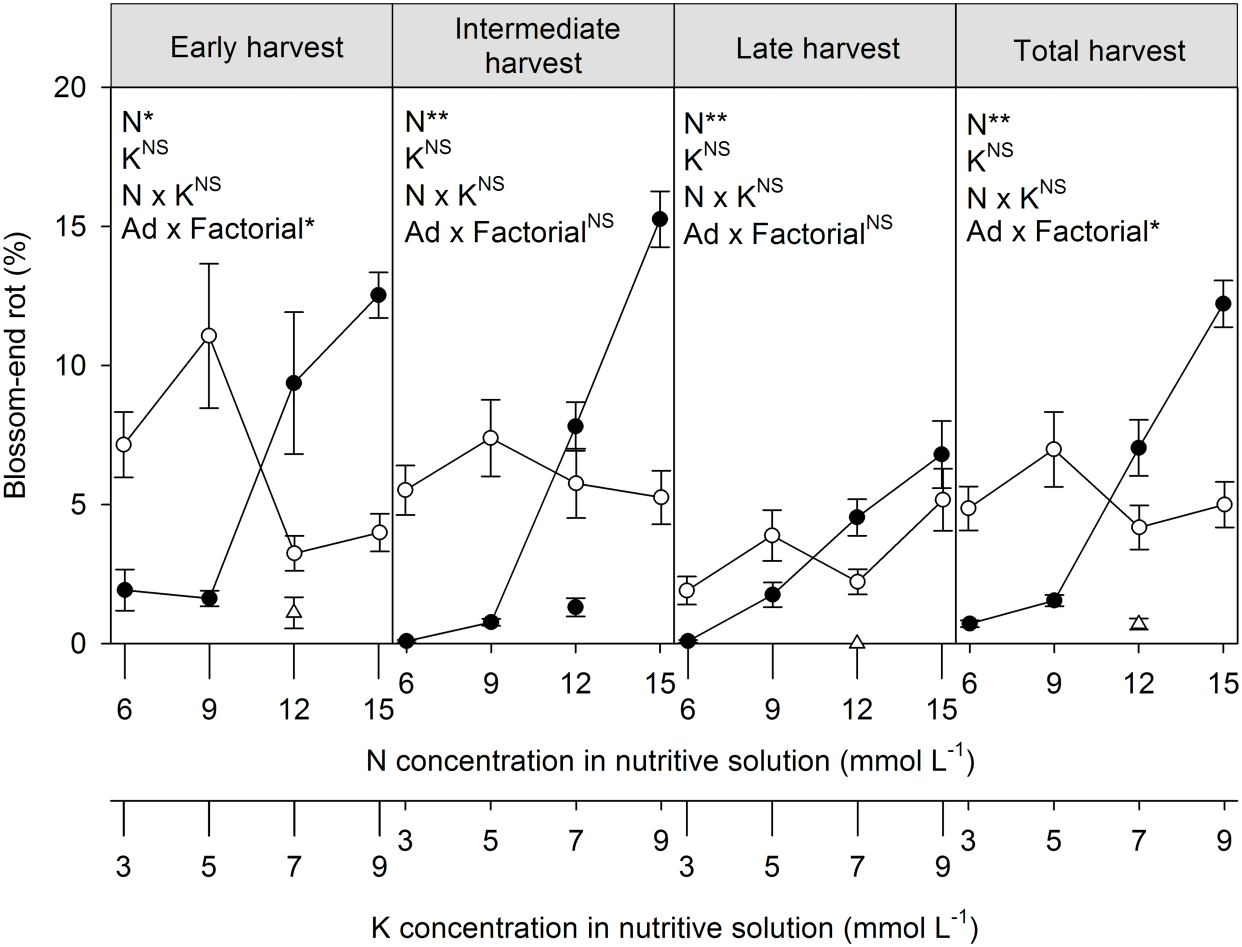
Percentage of fruits with blossom-end rot per evaluation period and total as a function of the nutrient-solution N (●) and K (○) concentration for bell pepper grown in the substrate without drainage. The triangle (Δ) indicates the average additional treatment (Ad) with 10–20% drainage of the nutrient-solution volume. Vertical bars represent the standard error. N, K, N x K and Ad x Factorial represent, in the analysis of variance, the causes of variation factor "concentration of N", factor "concentration of K", interaction N versus K, additional treatment (with drainage) versus the factorial treatments (without drainage), respectively. ^NS^, * and ** indicates F test not significant or significant at *p* = 0.05 or 0.01, respectively.

The percentage of fruit with blossom-end rot was not affected by the K concentration in the nutrient solution, and this variable was not influenced by the interaction between the N and K concentrations (Fig 3 and S4 Table). The percentage of fruits with blossom-end rot increased as the nutrient-solution N concentration increased from the 9 mmol L^-1^ in all harvest periods. The treatment with drainage had a lower percentage of fruits with blossom-end rot in the early harvest and in the total harvest relative to the treatments without drainage.

## Discussion

Potassium had little influence on pepper yield in a wide range of concentrations in the nutrient solution. Other studies have also reported no differences in bell-pepper yield at K concentrations ranging from 2.75 to 5.0 mmol L^-1^ [21], 3.0 to 6.0 mmol L^-1^ [21] and 2.5 to 7.0 mmol L^-1^ [22]. Concentrations of 1.5 mmol L^-1^ [21] and 14 mmol L^-1^ [22], however, decreased yield. Moreover, our results contrasted with those by Johnson and Decoteau [23], who concluded that at least 6.0 mmol L^-1^ K in the nutrient solution were required for optimal production. This concentration is similar to the recommendation of 6.3 mmol L^-1^ for hydroponics [15] used as a reference in our study.

The initial K concentration of 331.3 mg L^-1^ in the substrate may also have influenced the reduced need for K in bell-pepper cultivation without drainage. This K concentration, which is considered very high [24], is within the range of concentrations for most commercial coconut-coir substrates (115.8–2053.7 mg L^-1^). The high initial K concentration in the substrate may thus have contributed to the lack of effect of the nutrient-solution K concentrations on fruit yield. The nutrient-solution K concentrations also did not affect the amount of dry matter or the accumulation of K in the shoots, supporting this hypothesis (Table 2 and S2 Table). Furthermore, the substrate-solution K concentrations throughout the crop cycle were higher than the concentrations considered adequate, 12–45 mg L^-1^ [20], for all K levels analyzed (Fig 1 and S1 Table). Fruit weight and yield have responded positively to high nutrient-solution K concentrations using coconut-coir substrate and an initial K concentration of 24.8 mg L^-1^ without drainage in melon cultivation [10], indicating that the initial K concentration in the substrate can affect crop response to this nutrient.

The nutrient-solution N concentration that would maximize marketable fruit production was estimated at 10.7 mmol L^-1^, very similar to that recommended for the hydroponic cultivation of bell pepper of 11 mmol L^-1^ [15] used as a reference in our study (Fig 2 and S3 Table). The higher accumulation of N in the substrate from 132 DAP at nutrient-solution N concentrations >9 mmol L^-1^, however, indicated that these concentrations may be excessive after this time (Fig 1 and S1 Table). The N concentration of 6 mmol L^-1^ did not greatly increase the N concentration of the substrate during the culture of the pepper (Fig 1), i.e. the amount absorbed by the plants was similar to the amount provided by fertigation, indicating that concentrations below the recommended concentration of 11 mmol L^-1^ [15] may be the most suitable in the harvest period. The maximum yields of marketable fruits at the early, intermediate and late harvests at N concentrations of 12.5, 10 and 6 mmol L^-1^, respectively, also indicated that the nutrient-solution N concentration could be modified during the crop cycle.

Higher nutrient-solution N concentrations increased the number of fruits and was the main component influencing increased fruit yield up to 10.7 mmol L^-1^ (Fig 2 and S3 Table). These results were similar to those by Aminifard, Aroiee [25], who reported increases in the number of fruits per plant with increasing N concentrations. Higher nutrient-solution N concentrations, however, decreased fruit weight and was the main component that influenced the later decrease in fruit production from 10.7 mmol L^-1^. These results differed from those by Campos, Oliveira [26], who reported increases in fruit weight with increased N availability. The negative effect of N on fruit weight may thus have been due mainly to the increase in EC in the substrate solution (Fig 1 and S1 Table) rather than the actual direct effect of N. Rubio, Pereira [27] and Chartzoulakis and Klapaki [28] reported that increasing EC in the nutrient solution decreased fruit weight. Bell pepper is moderately sensitive to salinity, with an EC limit in soil crops of 1.5 dS m^-1^ (saturation extract), and plant production was estimated to decrease by 14% for every increase of 1.0 dS m^-1^ [29]. ECs between 0.6 and 1.5 dS m^-1^ are considered suitable for substrates (aqueous extraction at 1:1.5 v:v) [20]. The upper limit of 1.5 dS m^-1^ was exceeded at 132 DAP for the two highest nutrient-solution N concentrations (Fig 1 and S1 Table), which corresponds to the beginning of the intermediate harvest when fruit weight decreased more with increasing nutrient-solution N concentrations (Fig 2 and S3 Table). These results supported the effect of EC on the decrease in fruit yield with increasing nutrient-solution N concentrations from 10.7 mmol L^-1^. Likewise, the higher yield of fruits with blossom-end rot at the highest N concentration (Fig 3 and S4 Table) was likely associated more with the osmotic effect caused by the accumulation of N in the substrate. Rubio, Pereira [27] reported that increasing the EC of the nutrient solution increased the incidence of fruits with blossom-end rot.

K also increased the EC of the substrate above the limit of 1.5 dS m^-1^, initially at 132 DAP at the highest K concentration, but without causing losses in fruit yield and its components and without increasing the incidence of fruits with blossom-end rot. These results can be attributed to the effect of K in mitigating the negative effects of salt stress on the growth of bell pepper due to the maintenance of an adequate water status of the plants [30].

EC increased in the substrate solution (Fig 1 and S1 Table), even at the lowest nutrient-solution N and K concentrations, at which the accumulation of these nutrients in the substrate solution was lowest (Fig 3 and S4 Table), possibly due to the accumulation of other nutrients that were not assessed but were present in the nutrient solution. The increase in EC in the solution above the appropriate limit for a given culture has been associated with the rapid loss of fruit yield [29]. However, an increase in EC in the substrate was not enough to affect yield in short-cycle crops without drainage, such as melon in a coconut-coir substrate [10]. The accumulation of salts in the substrate due the lack of drainage for long-cycle crops such as bell pepper, however, is a factor that should be monitored and managed throughout the crop cycle to ensure the success of this system.

Increasing in the drainage percentage at ECs of the drained solution >1.0 dS m^-1^ relative to the EC of the nutrient solution is suggested for controlling EC in the substrate solution for cultivation with drainage [8]. Monitoring EC and the NO_3_^-^ and K^+^ ion concentrations of the substrate solution is likewise recommended for bell-pepper cultivation without drainage for reducing the nutrient-solution EC when the substrate-solution EC exceeds the limit of 1.5 dS m^-1^ (aqueous extraction at 1:1.5 (v:v)). Suction probes or syringes can be used for the collection of substrate-solution samples for monitoring EC [31], because the solution is not drained in this system. Obtaining, daily analysis and monitoring of these data over time is the main basis for the management of the nutrient solution in free-drainage crops [31] and should also be the basis in systems without drainage.

The higher fruit yield in the treatment with drainage than in those without drainage (Fig 2 and S3 Table) reinforced the need for excess fertigation to promote the renewal, and control the EC, of the substrate solution. The substrate-solution EC only exceeded the upper suitable limit of 1.5 dS m^-1^[20] in the last evaluation period, i.e. at the end of the cycle. Increasing the drainage percentage to avoid substrate salinization is nonetheless recommended when the substrate-solution EC exceeds this limit [8]. It is noteworthy that fruit yield per evaluation period was higher with (3.97 kg m^-2^) than without (mean of 2.83 kg m^-1^) drainage only in the late harvest, even though fruit weight had already been higher since the intermediate crop. Yield thus differed between the systems with (9.35 kg m^-2^) and without (mean of 8.00 kg m^-2^) drainage only at the final harvest, favoring the system with drainage. This result demonstrated the feasibility of the system without drainage, because its drawbacks will only will appear at the final phase of the cycle and can be managed.

## Conclusions

Bell-pepper cultivation in a fertigated substrate without drainage provided lower fruit production regardless of N and K concentrations compared to the recommended system with 10–20% drainage of the nutrient-solution volume. Higher nutrient-solution K concentrations did not affect plant production in the system without drainage for the coconut-coir substrate with an initial substrate K concentration of 331.3 mg L^-1^. Fruit yield increased at a nutrient-solution N concentration of 10.7 mmol L^-1^ in the system without drainage. However, concentrations of N in the nutrient solution of 13, 11 and 6 mmol L^-1^ would be more adequate during the early harvest, intermediate harvest and late harvest, respectively. The upper EC limit of the substrate solution was exceeded 181 days after planting in the system without drainage. In this situation, a decrease in the nutrient-solution nutrient concentrations or the use of drainage would be alternatives for controlling the substrate-solution EC.

## Supporting information

S1 Table EC in the substrate solution 61 (EC61), 132 (EC132), 181 (EC181) and 252 (EC252) days after planting (DAP); N concentration in the substrate solution 61 (N61), 132 (N132), 181 (N181) and 252 (N252) DAP and K concentration in the substrate solution 61 (K61), 132 (K132), 181 (K181) and 252 (K252) DAP. (DOCX)

S2 Table Dry mass and accumulation of N, K, P, Ca and Mg in the shoots. (DOCX)

S3 Table Bell-pepper marketable fruit yield (early harvest, MFYEH; intermediate harvest, MFYIH; late harvest, MFYLH and total harvest, MFYTH), number of marketable fruit (early harvest, NMFEH; intermediate harvest, NMFIH; late harvest, NMFLH and total harvest, NMFTH) and individual marketable fruit weight (early harvest, IMFWEH; intermediate harvest, IMFWIH; late harvest, IMFWLH and total harvest, IMFWTH). (DOCX)

S4 Table Percentage of blossom-end rot for the early harvest (BEREH), intermediate harvest (BERIH), late harvest (BERLH) and total harvest (BERTH). (DOCX)

## References

[1] Sanjuán MCS, Urrestarazu M. Métodos de riego y fertirrigación en cultivo sin suelo In: Urrestarazu M, editor. Tratado de cultivo sin suelo. 3 ed Madrid: Ediciones Mundi-Prensa; 2004 p. 161–237.

[2] Sonneveld C. La nutrición mineral y salinidad en los cultivos sin suelo: su manejo In: Urrestarazu M, editor. Tratado de cultivo sin suelo. 3 ed Madrid: Ediciones Mundi-Prensa; 2004 p. 305–67.

[3] Breś W. Estimation of nutrient losses from open fertigation systems to soil during horticultural plant cultivation. Polish J of Environ Stud. 2009;18(3):341–5.

[4] Massa D, Incrocci L, Maggini R, Carmassi G, Campiotti CA, Pardossi A. Strategies to decrease water drainage and nitrate emission from soilless cultures of greenhouse tomato. Agric Water Manag. 2010;97(7):971–80. doi: 10.1016/j.agwat.2010.01.029

[5] Muñoz P, Antón A, Paranjpe A, Ariño J, Montero JI. High decrease in nitrate leaching by lower N input without reducing greenhouse tomato yield. Agron Sustain Dev. 2008;28(4):489–95. doi: 10.1051/agro:2008024

[6] Thompson RB, Gallardo M, Rodríguez JS, Sánchez JA, Magán JJ. Effect of N uptake concentration on nitrate leaching from tomato grown in free-draining soilless culture under Mediterranean conditions. Sci Hortic (Amsterdam). 2013;150:387–98. doi: 10.1016/j.scienta.2012.11.018

[7] Urrestarazu M, Morales I, La Malfa T, Checa R, Wamser AF, Álvaro JE. Effects of Fertigation Duration on the Pollution, Water Consumption, and Productivity of Soilless Vegetable Cultures. HortScience. 2015;50(6):819–25.

[8] Miranda FR, Mesquita ALM, Martins MVV, Fernandes CMF, Evangelista MIP, Sousa AAP. Produção de tomate em substrato de fibra de coco. Fortaleza: EMBRAPA; 2011 20 p.

[9] Almeida OÁ. Qualidade da água de irrigação. Cruz das Almas: Embrapa Mandioca e Fruticultura; 2010 234 p.

[10] Gratieri LA, Filho ABC, Barbosa JC, Pavani LC. Nitrogen and potassium concentrations in the nutrients solution for melon plants growing in coconut fiber without drainage. Scientific World Journal. 2013;2013:1–10. doi: 10.1155/2013/546594 23864827 10.1155/2013/546594 PMC3705887

[11] Choi E-Y, Seo S-K, Choi K-Y, Lee Y-B. Development of a non-drainage hydroponic system with a coconut coir substrate by a frequency domain reflectometry sensor for tomato cultivation. J Plant Nutr. 2014;37(5):748–64. doi: 10.1080/01904167.2013.868479

[12] Henz GP, Ribeiro CS, Carvalho SIC, Banci CA. Como cultivar pimentão. Revista Cultivar HF. 2007;42:2–7.

[13] Charlo HCO, Oliveira SF, Vargas PF, Castoldi R, Barbosa JC, Braz LT. Accumulation of nutrients in sweet peppers cultivated in coconut fiber. Hortic Bras. 2012;30(1):125–31. doi: 10.1590/S0102-05362012000100021

[14] Sonneveld C, Elderen CW. Chemical analysis of peaty growing media by means of water extraction. Commun Soil Sci Plant Anal. 1994;25(19–20):3199–208. doi: 10.1080/00103629409369258

[15] Castellane PD, Araujo JAC. Cultivo sem solo: hidroponia. Jaboticabal: FUNEP; 1994.

[16] Calbo AG, Silva WLC. Sistema Irrigas para manejo de irrigação: fundamentos, aplicações e desenvolvimentos. Brasília: Embrapa Hortaliças; 2005 174 p.

[17] Bataglia OC, Furlani AMC, Teixeira JPF, Furlani PR, Gallo JR. Métodos de análise química de plantas. Campinas: Instituto Agronômico; 1983 48 p.

[18] Ferreira EB, Cavalcanti PP, Nogueira DA, editors. Função para analisar experimentos em fatorial duplo com um tratamento adicional, em uma só rodada. Congresso de Pós-Graduação da Universidade Federal de Lavras, 19; 2010; Lavras: UFLA.

[19] Healy MJR. The analysis of a factorial experiment with additional treatments. Journal of Agricultural Science. 1956;47(2):205–6. doi: 10.1017/S0021859600040120

[20] Pardossi A, Carmassi G, Diara C, Incrocci L, Maggini R, Massa D. Fertigation and Substrate Management in Closed Soilless Culture. Pisa: University of Pisa; 2011 63 p.

[21] Lozano MG, Escobar I, Berenguer JJ. Green-pepper fertigation in soilless culture. Acta Hortic. 2005;697:543–7. doi: 10.17660/ActaHortic.2005.697.71

[22] Rubio JS, García-Sánchez F, Flores P, Navarro JM, Martínez V. Yield and fruit quality of sweet pepper in response to fertilisation with Ca2+ and K+. Span J Agric Res. 2010;8(1):170–7. doi: 10.5424/sjar/2010081-1156

[23] Johnson CD, Decoteau DR. Nitrogen and potassium fertility affects jalapeño pepper plant growth, pod yield, and pungency. HortScience. 1996;31(7):1119–23.

[24] Abad M, Noguera P, Puchades R, Maquieira A, Noguera V. Physico-chemical and chemical properties of some coconut coir dusts for use as a peat substitute for containerised ornamental plants. Bioresour Technol. 2002;82(3):241–5. doi: 10.1016/S0960-8524(01)00189-4 11991072 10.1016/s0960-8524(01)00189-4

[25] Aminifard MH, Aroiee H, Nemati H, Azizi M, Khayyat M. Effect of nitrogen fertilizer on vegetative and reproductive growth of pepper plants under field conditions. J Plant Nutr. 2012;35(2):235–42. doi: 10.1080/01904167.2012.636126

[26] Campos VB, Oliveira APd, Cavalcante LF, Prazeres SdS. Rendimento do pimentão submetido ao nitrogênio aplicado via água de irrigação em ambiente protegido. Revista de Biologia e Ciências da Terra 2008;8(2):72–9.

[27] Rubio JS, Pereira WE, Garcia-Sanchez F, Murillo L, García AL, Martínez V. Sweet pepper production in substrate in response to salinity, nutrient solution management and training system. Hortic Bras. 2011;29(3):275–81. doi: 10.1590/S0102-05362011000300003

[28] Chartzoulakis K, Klapaki G. Response of two greenhouse pepper hybrids to NaCl salinity during different growth stages. Sci Hortic (Amsterdam). 2000;86(3):247–60. doi: 10.1016/S0304-4238(00)00151-5

[29] Hanson BR, Grattan SR, Fulton A. Agricultural salinity and drainage. Davis: University of California; 2006 164 p.

[30] Rubio JS, García-Sánchez F, Rubio F, García AL, Martínez V. The Importance of K+ in ameliorating the negative effects of salt stress on the growth of pepper plants. Eur J Hortic Sci. 2010;75(1):33–41.

[31] Soria CB, Olivert JMA. Cultivo sin suelo de hortalizas: aspectos prácticos y experiencias. Valencia: Generalitat Valenciana; 2002 111 p.

