# Supplementary material for: Influence of drainage and nutrient-solution nitrogen and potassium concentrations on the agronomic behavior of bell-pepper plants cultivated in a substrate
Source: PLoS One. 2017 Jul 5;12(7):e0180529. doi: 10.1371/journal.pone.0180529 (PMC5498029; doi:10.1371/journal.pone.0180529)
Supplement: S1 Table — (DOCX) [file pone.0180529.s001.docx]

**S1 Table. EC in the substrate solution 61 (EC61), 132 (EC132), 181 (EC181) and 252 (EC252) days after planting (DAP); N concentration in the substrate solution 61 (N61), 132 (N132), 181 (N181) and 252 (N252) DAP and K concentration in the substrate solution 61 (K61), 132 (K132), 181 (K181) and 252 (K252) DAP**

| **N concentration** | **K concentration** | **Replicate** | **EC61** | **EC132** | **EC181** | **EC252** | **N61** | **N132** | **N181** | **N252** | **K61** | **K132** | **K181** | **K252** |
| --- | --- | --- | --- | --- | --- | --- | --- | --- | --- | --- | --- | --- | --- | --- |
| 6 | 3 | 1 | 0.58 | 0.82 | 1.26 | 0.68 | 10.1 | 11.6 | 15.4 | 6.0 | 177.4 | 172.5 | 154.3 | 129,5 |
| 6 | 3 | 2 | 0.89 | 0.51 | 1.36 | 0.86 | 11.6 | 17.4 | 21.6 | 26.8 | 278.0 | 83.7 | 176.9 | 132,1 |
| 6 | 3 | 3 | 0.88 | 0.60 | 0.84 | 1.15 | 11.6 | 12.3 | 10.8 | 13.1 | 274.7 | 106.9 | 181.1 | 197,8 |
| 6 | 5 | 1 | 1.33 | 1.09 | 1.51 | 2.14 | 28.6 | 8.7 | 13.7 | 93.9 | 362.5 | 269.8 | 321.5 | 338,3 |
| 6 | 5 | 2 | 1.03 | 1.08 | 1.70 | 3.59 | 10.1 | 4.7 | 15.7 | 24.2 | 357.5 | 312.8 | 361.9 | 536,5 |
| 6 | 5 | 3 | 0.60 | 1.12 | 1.63 | 1.62 | 10.1 | 22.1 | 16.0 | 24.9 | 176.2 | 274.4 | 277.4 | 307,3 |
| 6 | 7 | 1 | 0.63 | 1.03 | 1.57 | 1.22 | 11.6 | 9.8 | 14.1 | 20.0 | 200.0 | 275.3 | 390.3 | 281,7 |
| 6 | 7 | 2 | 0.90 | 1.14 | 1.59 | 1.15 | 11.6 | 6.9 | 14.1 | 23.6 | 288.9 | 275.6 | 295.9 | 292,9 |
| 6 | 7 | 3 | 0.64 | 0.95 | 1.35 | 1.42 | 6.9 | 16.3 | 14.1 | 23.6 | 193.9 | 212.6 | 265.3 | 289,9 |
| 6 | 9 | 1 | 0.96 | 1.22 | 2.01 | 2.17 | 9.4 | 4.3 | 43.8 | 26.8 | 307.1 | 367.3 | 464.5 | 509,7 |
| 6 | 9 | 2 | 1.04 | 1.11 | 2.06 | 1.18 | 13.4 | 13.4 | 30.8 | 20.3 | 304.9 | 292.1 | 489.7 | 329,6 |
| 6 | 9 | 3 | 0.91 | 1.04 | 1.63 | 2.09 | 8.0 | 12.7 | 13.7 | 24.9 | 289.5 | 300.8 | 348.3 | 482,3 |
| 9 | 3 | 1 | 0.88 | 0.98 | 1.11 | 0.98 | 12.7 | 14.1 | 41.2 | 46.8 | 277.5 | 227.5 | 196.9 | 191,0 |
| 9 | 3 | 2 | 0.71 | 1.00 | 1.27 | 2.17 | 10.9 | 10.9 | 25.2 | 72.6 | 222.2 | 259.0 | 153.4 | 242,8 |
| 9 | 3 | 3 | 0.47 | 1.02 | 0.83 | 1.22 | 12.5 | 18.5 | 25.2 | 47.8 | 132.5 | 189.9 | 166.1 | 190,3 |
| 9 | 5 | 1 | 0.96 | 1.63 | 2.22 | 2.15 | 10.9 | 20.2 | 80.5 | 113.2 | 306.6 | 353.7 | 408.7 | 379,3 |
| 9 | 5 | 2 | 1.19 | 1.16 | 1.53 | 1.96 | 12.3 | 22.5 | 36.0 | 74.6 | 357.7 | 349.7 | 323.5 | 400,0 |
| 9 | 5 | 3 | 0.76 | 1.00 | 1.47 | 1.78 | 19.9 | 23.2 | 54.3 | 77.2 | 237.2 | 231.8 | 340.9 | 284,6 |
| 9 | 7 | 1 | 1.03 | 1.42 | 1.46 | 1.28 | 15.2 | 38.4 | 67.7 | 42.2 | 319.4 | 362.5 | 354.1 | 303,8 |
| 9 | 7 | 2 | 0.90 | 1.29 | 1.64 | 1.25 | 12.3 | 18.5 | 29.1 | 41.2 | 289.3 | 361.2 | 408.5 | 313,8 |
| 9 | 7 | 3 | 0.67 | 0.94 | 2.06 | 2.54 | 9.8 | 35.1 | 53.0 | 66.7 | 211.5 | 242.7 | 417.8 | 502,6 |
| 9 | 9 | 1 | 0.76 | 1.92 | 2.75 | 2.13 | 6.9 | 15.2 | 90.3 | 74.6 | 279.5 | 497.5 | 664.3 | 484,2 |
| 9 | 9 | 2 | 0.91 | 1.23 | 3.23 | 2.12 | 26.8 | 32.6 | 52.3 | 59.5 | 271.5 | 337.4 | 587.1 | 496,3 |
| 9 | 9 | 3 | 0.94 | 1.65 | 1.10 | 1.93 | 18.1 | 45.6 | 36.3 | 60.2 | 291.6 | 424.0 | 277.4 | 548,0 |
| 12 | 3 | 1 | 1.44 | 0.38 | 0.47 | 1.01 | 13.4 | 32.9 | 57.2 | 19.6 | 372.7 | 83.3 | 85.9 | 219,4 |
| 12 | 3 | 2 | 0.98 | 1.59 | 3.36 | 2.62 | 10.5 | 66.2 | 200.8 | 198.2 | 311.8 | 224.5 | 386.8 | 224,7 |
| 12 | 3 | 3 | 0.61 | 1.82 | 1.65 | 1.90 | 7.6 | 79.6 | 111.9 | 133.5 | 193.1 | 251.4 | 151.7 | 138,4 |
| 12 | 5 | 1 | 0.84 | 1.44 | 2.00 | 1.99 | 9.4 | 65.5 | 121.0 | 149.2 | 281.6 | 294.7 | 375.2 | 333,4 |
| 12 | 5 | 2 | 1.19 | 1.77 | 2.31 | 1.77 | 38.7 | 73.8 | 126.9 | 105.3 | 292.9 | 262.6 | 422.2 | 242,8 |
| 12 | 5 | 3 | 0.99 | 1.74 | 2.79 | 3.23 | 15.9 | 76.7 | 172.1 | 193.6 | 298.0 | 339.3 | 459.8 | 462,7 |
| 12 | 7 | 1 | 0.99 | 1.31 | 1.64 | 0.87 | 9.0 | 50.3 | 118.4 | 38.9 | 321.7 | 350.5 | 377.6 | 273,2 |
| 12 | 7 | 2 | 0.89 | 1.56 | 1.12 | 3.24 | 9.4 | 47.8 | 30.8 | 161.6 | 288.1 | 432.3 | 340.1 | 668,1 |
| 12 | 7 | 3 | 0.93 | 1.61 | 1.50 | 2.80 | 20.3 | 88.7 | 94.2 | 184.5 | 290.8 | 387.0 | 382.9 | 584,0 |
| 12 | 9 | 1 | 1.44 | 2.05 | 1.88 | 1.58 | 24.6 | 98.4 | 128.9 | 89.0 | 405.3 | 491.8 | 437.1 | 379,7 |
| 12 | 9 | 2 | 0.98 | 1.42 | 2.33 | 2.97 | 10.1 | 29.7 | 116.5 | 141.3 | 313.9 | 426.6 | 635.4 | 688,3 |
| 12 | 9 | 3 | 0.78 | 1.78 | 3.27 | 3.03 | 13.0 | 66.2 | 177.9 | 210.0 | 252.7 | 470.1 | 705.8 | 613,7 |
| 15 | 3 | 1 | 0.89 | 1.65 | 3.58 | 1.90 | 26.1 | 97.3 | 306.8 | 143.3 | 264.2 | 227.7 | 306.3 | 218,7 |
| 15 | 3 | 2 | 0.71 | 1.89 | 2.67 | 1.73 | 10.1 | 115.4 | 208.0 | 121.0 | 250.2 | 284.7 | 383.2 | 174,0 |
| 15 | 3 | 3 | 0.74 | 1.81 | 1.25 | 1.26 | 32.2 | 129.9 | 110.2 | 107.6 | 194.7 | 169.1 | 137.1 | 157,0 |
| 15 | 5 | 1 | 1.17 | 1.76 | 1.99 | 1.78 | 34.0 | 101.0 | 140.7 | 139.3 | 320.4 | 317.7 | 374.3 | 257,6 |
| 15 | 5 | 2 | 1.01 | 2.15 | 1.70 | 2.48 | 12.7 | 99.2 | 126.9 | 215.2 | 318.6 | 425.5 | 324.4 | 407,9 |
| 15 | 5 | 3 | 0.76 | 0.94 | 1.08 | 1.89 | 33.3 | 65.9 | 114.5 | 144.6 | 215.2 | 166.6 | 171.9 | 260,1 |
| 15 | 7 | 1 | 1.34 | 1.92 | 3.46 | 2.31 | 29.0 | 112.5 | 245.3 | 154.4 | 383.9 | 415.0 | 647.8 | 399,1 |
| 15 | 7 | 2 | 0.97 | 2.03 | 2.99 | 2.59 | 37.3 | 120.9 | 263.6 | 154.4 | 282.0 | 421.7 | 525.8 | 459,9 |
| 15 | 7 | 3 | 1.05 | 2.00 | 2.74 | 1.72 | 81.1 | 121.6 | 183.8 | 118.4 | 258.4 | 395.9 | 575.0 | 348,2 |
| 15 | 9 | 1 | 1.05 | 2.47 | 2.38 | 3.49 | 15.9 | 178.8 | 200.8 | 251.2 | 337.3 | 577.2 | 529.5 | 706,0 |
| 15 | 9 | 2 | 1.09 | 2.23 | 4.00 | 2.61 | 15.2 | 127.4 | 321.2 | 212.6 | 349.3 | 589.0 | 876.2 | 542,6 |
| 15 | 9 | 3 | 0.79 | 1.34 | 3.08 | 2.15 | 12.3 | 80.7 | 292.4 | 172.7 | 282.8 | 359.4 | 639.8 | 456,6 |
| Additional treatment | | 1 | 0,65 | 1.33 | 1.40 | 2.00 | 26.8 | 81.1 | 89.6 | 150.6 | 202.7 | 351.0 | 498.2 | 508.2 |
| Additional treatment | | 2 | 0,63 | 1.17 | 1.49 | 2.14 | 10.9 | 54.6 | 68.9 | 180.0 | 229.1 | 308.7 | 413.9 | 558.4 |
| Additional treatment | | 3 | 0,58 | 1.40 | 1.30 | 1.27 | 9.4 | 85.4 | 85.5 | 128.0 | 184.5 | 388.3 | 287.4 | 301.3 |
