# Supplementary material for: Influence of drainage and nutrient-solution nitrogen and potassium concentrations on the agronomic behavior of bell-pepper plants cultivated in a substrate
Source: PLoS One. 2017 Jul 5;12(7):e0180529. doi: 10.1371/journal.pone.0180529 (PMC5498029; doi:10.1371/journal.pone.0180529)
Supplement: S2 Table — (DOCX) [file pone.0180529.s002.docx]

**S2 Table. Dry mass and accumulation of N, K, P, Ca and Mg in the shoots.**

| **N concentration** | **K concentration** | **Replicate** | **Dry mass** | **N** | **K** | **P** | **Ca** | **Mg** |
| --- | --- | --- | --- | --- | --- | --- | --- | --- |
| 6 | 3 | 1 | 595.70 | 14.43 | 20.04 | 3.37 | 3.86 | 1.84 |
| 6 | 3 | 2 | 458.09 | 10.67 | 15.79 | 2.60 | 2.40 | 1.18 |
| 6 | 3 | 3 | 447.77 | 11.58 | 14.85 | 2.76 | 2.36 | 1.17 |
| 6 | 5 | 1 | 469.41 | 13.22 | 16.09 | 2.67 | 2.93 | 1.52 |
| 6 | 5 | 2 | 370.35 | 10.40 | 11.94 | 2.19 | 1.97 | 0.87 |
| 6 | 5 | 3 | 365.44 | 9.86 | 12.89 | 2.17 | 1.97 | 0.89 |
| 6 | 7 | 1 | 357.55 | 8.26 | 11.98 | 2.11 | 1.47 | 0.82 |
| 6 | 7 | 2 | 439.51 | 11.64 | 15.82 | 2.52 | 2.48 | 1.15 |
| 6 | 7 | 3 | 418.61 | 9.82 | 10.63 | 2.37 | 2.21 | 0.87 |
| 6 | 9 | 1 | 459.33 | 11.37 | 16.01 | 2.44 | 2.38 | 1.18 |
| 6 | 9 | 2 | 370.73 | 9.72 | 13.04 | 1.90 | 1.76 | 0.92 |
| 6 | 9 | 3 | 484.86 | 13.19 | 17.38 | 2.67 | 3.00 | 1.33 |
| 9 | 3 | 1 | 430.19 | 10.52 | 14.12 | 2.67 | 2.08 | 1.10 |
| 9 | 3 | 2 | 502.23 | 12.57 | 17.10 | 3.26 | 2.47 | 1.30 |
| 9 | 3 | 3 | 532.10 | 13.60 | 20.49 | 3.28 | 3.17 | 1.54 |
| 9 | 5 | 1 | 580.92 | 12.72 | 19.89 | 3.33 | 2.90 | 1.53 |
| 9 | 5 | 2 | 580.67 | 11.86 | 19.26 | 3.01 | 2.83 | 1.53 |
| 9 | 5 | 3 | 433.63 | 10.85 | 15.33 | 2.44 | 2.37 | 1.30 |
| 9 | 7 | 1 | 353.57 | 9.14 | 12.03 | 1.79 | 1.40 | 0.87 |
| 9 | 7 | 2 | 487.40 | 12.00 | 17.23 | 2.78 | 2.08 | 1.28 |
| 9 | 7 | 3 | 445.06 | 9.85 | 16.12 | 1.57 | 2.12 | 1.13 |
| 9 | 9 | 1 | 616.16 | 16.38 | 21.41 | 3.89 | 3.10 | 1.74 |
| 9 | 9 | 2 | 517.46 | 12.79 | 18.16 | 3.34 | 2.32 | 1.23 |
| 9 | 9 | 3 | 673.01 | 18.03 | 23.35 | 4.38 | 3.54 | 1.86 |
| 12 | 3 | 1 | 313.92 | 9.09 | 10.70 | 1.77 | 1.65 | 0.82 |
| 12 | 3 | 2 | 503.39 | 13.96 | 16.81 | 2.67 | 2.13 | 1.24 |
| 12 | 3 | 3 | 488.40 | 14.17 | 17.19 | 2.78 | 1.96 | 1.14 |
| 12 | 5 | 1 | 513.57 | 12.91 | 17.19 | 2.80 | 2.05 | 1.30 |
| 12 | 5 | 2 | 468.22 | 13.41 | 16.68 | 2.21 | 3.17 | 1.65 |
| 12 | 5 | 3 | 564.43 | 15.59 | 20.29 | 2.96 | 2.70 | 1.56 |
| 12 | 7 | 1 | 514.35 | 14.02 | 17.77 | 2.75 | 3.04 | 1.52 |
| 12 | 7 | 2 | 566.02 | 15.81 | 19.93 | 3.51 | 2.53 | 1.58 |
| 12 | 7 | 3 | 543.36 | 14.82 | 18.24 | 2.93 | 2.46 | 1.44 |
| 12 | 9 | 1 | 418.14 | 9.53 | 14.26 | 2.30 | 1.92 | 1.02 |
| 12 | 9 | 2 | 543.86 | 13.03 | 18.83 | 3.10 | 2.47 | 1.36 |
| 12 | 9 | 3 | 506.44 | 11.55 | 18.01 | 2.88 | 2.59 | 1.44 |
| 15 | 3 | 1 | 583.44 | 18.63 | 19.47 | 3.27 | 3.33 | 1.76 |
| 15 | 3 | 2 | 368.66 | 10.87 | 11.75 | 2.28 | 1.20 | 0.81 |
| 15 | 3 | 3 | 564.55 | 16.45 | 19.10 | 3.23 | 2.31 | 1.43 |
| 15 | 5 | 1 | 422.76 | 10.95 | 14.87 | 2.40 | 2.23 | 1.06 |
| 15 | 5 | 2 | 486.45 | 11.57 | 17.73 | 2.57 | 2.59 | 1.25 |
| 15 | 5 | 3 | 560.53 | 14.15 | 20.36 | 3.29 | 2.74 | 1.45 |
| 15 | 7 | 1 | 462.04 | 11.06 | 15.73 | 2.39 | 2.36 | 1.08 |
| 15 | 7 | 2 | 605.28 | 16.20 | 20.65 | 3.24 | 3.25 | 1.61 |
| 15 | 7 | 3 | 468.41 | 12.29 | 16.42 | 2.53 | 2.63 | 1.35 |
| 15 | 9 | 1 | 471.16 | 12.99 | 16.30 | 2.64 | 2.15 | 1.22 |
| 15 | 9 | 2 | 428.17 | 11.02 | 14.22 | 2.47 | 1.52 | 1.02 |
| 15 | 9 | 3 | 603.79 | 16.22 | 21.24 | 3.25 | 2.97 | 1.69 |
| Additional treatment | | 1 | 532,42 | 12.70 | 18.75 | 3.19 | 2.44 | 1.41 |
| Additional treatment | | 2 | 633,71 | 15.72 | 23.73 | 3.32 | 3.19 | 1.88 |
| Additional treatment | | 3 | 540,26 | 13.13 | 20.12 | 3.08 | 2.61 | 1.40 |
