# Supplementary material for: Influence of drainage and nutrient-solution nitrogen and potassium concentrations on the agronomic behavior of bell-pepper plants cultivated in a substrate
Source: PLoS One. 2017 Jul 5;12(7):e0180529. doi: 10.1371/journal.pone.0180529 (PMC5498029; doi:10.1371/journal.pone.0180529)
Supplement: S3 Table — (DOCX) [file pone.0180529.s003.docx]

**S3 Table. Bell-pepper marketable fruit yield (early harvest, MFYEH; intermediate harvest, MFYIH; late harvest, MFYLH and total harvest, MFYTH), number of marketable fruit (early harvest, NMFEH; intermediate harvest, NMFIH; late harvest, NMFLH and total harvest, NMFTH) and individual marketable fruit weight (early harvest, IMFWEH; intermediate harvest, IMFWIH; late harvest, IMFWLH and total harvest, IMFWTH).**

| **N concentration** | **K concentration** | **Replicate** | **MFYEH** | **MFYIH** | **MFYLH** | **MFYTH** | **NMFEH** | **NMFIH** | **NMFLH** | **NMFTH** | **IMFWEH** | **IMFWIH** | **IMFWLH** | **IMFWTH** |
| --- | --- | --- | --- | --- | --- | --- | --- | --- | --- | --- | --- | --- | --- | --- |
| 6 | 3 | 1 | 1.711 | 3.689 | 2.885 | 8.285 | 9.7 | 17.0 | 15.5 | 42.2 | 177.0 | 217.0 | 186.1 | 196.5 |
| 6 | 3 | 2 | 1.764 | 2.934 | 3.623 | 8.322 | 10.7 | 14.7 | 18.7 | 44.2 | 164.4 | 199.2 | 193.5 | 188.3 |
| 6 | 3 | 3 | 1.666 | 2.934 | 3.048 | 7.648 | 10.2 | 15.3 | 14.9 | 40.4 | 163.6 | 192.1 | 204.5 | 189.5 |
| 6 | 5 | 1 | 0.592 | 3.368 | 3.118 | 7.079 | 5.1 | 21.3 | 19.6 | 46.0 | 115.9 | 157.9 | 159.5 | 153.9 |
| 6 | 5 | 2 | 1.161 | 2.878 | 2.867 | 6.907 | 7.8 | 14.7 | 14.2 | 36.7 | 148.5 | 195.4 | 202.1 | 188.0 |
| 6 | 5 | 3 | 1.444 | 2.674 | 2.741 | 6.859 | 9.0 | 13.0 | 13.2 | 35.2 | 160.5 | 205.7 | 208.1 | 195.0 |
| 6 | 7 | 1 | 1.624 | 2.899 | 2.957 | 7.481 | 9.8 | 14.7 | 16.5 | 41.1 | 165.4 | 196.9 | 178.7 | 182.1 |
| 6 | 7 | 2 | 1.684 | 3.021 | 2.733 | 7.437 | 10.3 | 16.0 | 14.3 | 40.7 | 163.0 | 188.8 | 190.6 | 182.9 |
| 6 | 7 | 3 | 1.892 | 2.677 | 2.872 | 7.441 | 11.3 | 13.7 | 15.0 | 40.0 | 167.0 | 195.9 | 191.4 | 186.0 |
| 6 | 9 | 1 | 1.391 | 3.035 | 3.241 | 7.667 | 8.6 | 15.4 | 19.4 | 43.4 | 161.8 | 197.1 | 167.0 | 176.7 |
| 6 | 9 | 2 | 1.605 | 2.903 | 2.694 | 7.203 | 9.5 | 15.7 | 14.7 | 39.8 | 169.0 | 185.3 | 183.7 | 180.8 |
| 6 | 9 | 3 | 1.458 | 2.806 | 3.278 | 7.541 | 9.5 | 14.4 | 17.5 | 41.3 | 154.2 | 195.3 | 187.8 | 182.7 |
| 9 | 3 | 1 | 1.862 | 3.262 | 2.398 | 7.522 | 10.5 | 17.3 | 15.5 | 43.3 | 176.6 | 188.9 | 155.1 | 173.8 |
| 9 | 3 | 2 | 2.433 | 3.196 | 3.098 | 8.726 | 13.3 | 15.0 | 15.3 | 43.7 | 182.4 | 213.0 | 202.0 | 199.8 |
| 9 | 3 | 3 | 2.152 | 3.570 | 3.684 | 9.405 | 10.6 | 17.2 | 18.2 | 46.0 | 203.0 | 207.5 | 202.4 | 204.5 |
| 9 | 5 | 1 | 2.245 | 3.704 | 3.580 | 9.530 | 13.1 | 18.5 | 19.6 | 51.3 | 171.5 | 199.7 | 182.3 | 185.9 |
| 9 | 5 | 2 | 1.695 | 3.532 | 3.043 | 8.270 | 11.3 | 19.5 | 17.8 | 48.5 | 150.6 | 181.1 | 171.5 | 170.5 |
| 9 | 5 | 3 | 1.165 | 2.099 | 3.432 | 6.696 | 7.1 | 9.8 | 15.6 | 32.5 | 164.3 | 213.8 | 219.5 | 205.7 |
| 9 | 7 | 1 | 1.603 | 2.946 | 1.891 | 6.440 | 9.1 | 20.0 | 12.5 | 41.6 | 176.4 | 147.3 | 150.7 | 154.7 |
| 9 | 7 | 2 | 2.071 | 4.144 | 3.006 | 9.221 | 12.0 | 19.8 | 19.4 | 51.2 | 172.6 | 209.3 | 154.9 | 180.1 |
| 9 | 7 | 3 | 2.638 | 3.182 | 2.833 | 8.653 | 14.9 | 16.2 | 15.5 | 46.5 | 177.0 | 196.6 | 183.3 | 185.9 |
| 9 | 9 | 1 | 2.246 | 3.729 | 3.148 | 9.123 | 12.7 | 19.3 | 19.2 | 51.2 | 177.3 | 192.9 | 164.2 | 178.3 |
| 9 | 9 | 2 | 2.243 | 3.367 | 2.866 | 8.476 | 13.1 | 17.5 | 16.5 | 47.1 | 171.3 | 192.9 | 173.2 | 180.0 |
| 9 | 9 | 3 | 2.630 | 4.336 | 4.220 | 11.187 | 13.0 | 22.4 | 20.4 | 55.8 | 202.3 | 193.6 | 206.9 | 200.5 |
| 12 | 3 | 1 | 1.398 | 2.573 | 1.460 | 5.430 | 8.0 | 18.0 | 9.5 | 35.5 | 174.7 | 142.9 | 153.7 | 153.0 |
| 12 | 3 | 2 | 2.340 | 3.317 | 2.972 | 8.629 | 12.2 | 17.8 | 16.2 | 46.2 | 192.1 | 186.2 | 183.7 | 186.9 |
| 12 | 3 | 3 | 2.527 | 2.958 | 3.290 | 8.775 | 13.0 | 15.8 | 18.2 | 47.0 | 194.4 | 186.8 | 181.1 | 186.7 |
| 12 | 5 | 1 | 2.481 | 3.630 | 2.471 | 8.582 | 13.3 | 20.5 | 13.8 | 47.6 | 186.9 | 176.7 | 178.8 | 180.2 |
| 12 | 5 | 2 | 0.511 | 2.972 | 2.615 | 6.099 | 4.0 | 18.7 | 15.5 | 38.2 | 127.9 | 158.7 | 169.2 | 159.7 |
| 12 | 5 | 3 | 2.391 | 3.552 | 3.185 | 9.128 | 13.0 | 20.4 | 18.0 | 51.4 | 183.9 | 174.1 | 176.9 | 177.6 |
| 12 | 7 | 1 | 2.520 | 3.223 | 2.198 | 7.940 | 14.2 | 18.5 | 14.5 | 47.3 | 177.7 | 173.8 | 151.1 | 168.0 |
| 12 | 7 | 2 | 2.590 | 3.546 | 2.706 | 8.842 | 13.8 | 19.4 | 14.8 | 48.0 | 187.7 | 182.8 | 182.8 | 184.2 |
| 12 | 7 | 3 | 2.583 | 3.106 | 3.151 | 8.839 | 13.7 | 15.0 | 17.5 | 46.2 | 189.0 | 207.0 | 180.1 | 191.5 |
| 12 | 9 | 1 | 2.163 | 2.708 | 2.547 | 7.417 | 12.8 | 19.2 | 18.0 | 50.0 | 168.5 | 141.3 | 141.5 | 148.3 |
| 12 | 9 | 2 | 2.002 | 3.940 | 2.742 | 8.684 | 11.4 | 22.4 | 16.8 | 50.6 | 175.6 | 175.9 | 163.2 | 171.6 |
| 12 | 9 | 3 | 2.158 | 3.202 | 2.895 | 8.255 | 10.5 | 17.0 | 16.8 | 44.3 | 205.5 | 188.4 | 172.0 | 186.2 |
| 15 | 3 | 1 | 2.287 | 3.850 | 2.576 | 8.713 | 12.4 | 23.8 | 16.5 | 52.7 | 185.0 | 161.6 | 155.7 | 165.2 |
| 15 | 3 | 2 | 1.927 | 2.823 | 2.356 | 7.106 | 11.8 | 17.8 | 13.2 | 42.8 | 162.9 | 158.3 | 178.9 | 165.9 |
| 15 | 3 | 3 | 2.486 | 3.282 | 3.449 | 9.217 | 14.4 | 18.9 | 19.6 | 52.9 | 172.1 | 173.8 | 176.4 | 174.3 |
| 15 | 5 | 1 | 2.086 | 2.927 | 1.834 | 6.847 | 12.2 | 20.9 | 12.4 | 45.5 | 171.2 | 140.0 | 148.3 | 150.6 |
| 15 | 5 | 2 | 2.070 | 3.396 | 2.049 | 7.515 | 12.3 | 23.0 | 14.0 | 49.3 | 167.8 | 147.7 | 146.4 | 152.3 |
| 15 | 5 | 3 | 2.392 | 3.063 | 3.442 | 8.897 | 13.0 | 16.7 | 18.8 | 48.5 | 184.0 | 183.8 | 182.7 | 183.4 |
| 15 | 7 | 1 | 2.263 | 2.871 | 2.281 | 7.416 | 14.0 | 20.4 | 14.2 | 48.5 | 161.7 | 141.0 | 160.8 | 152.8 |
| 15 | 7 | 2 | 2.009 | 3.294 | 2.567 | 7.870 | 11.8 | 19.8 | 14.5 | 46.2 | 170.0 | 166.2 | 176.5 | 170.4 |
| 15 | 7 | 3 | 2.526 | 2.797 | 2.024 | 7.346 | 14.2 | 16.8 | 11.8 | 42.8 | 177.9 | 166.5 | 171.5 | 171.6 |
| 15 | 9 | 1 | 2.030 | 2.981 | 2.438 | 7.449 | 12.2 | 19.8 | 17.2 | 49.2 | 166.4 | 150.6 | 141.7 | 151.4 |
| 15 | 9 | 2 | 2.050 | 2.888 | 2.465 | 7.403 | 12.9 | 19.5 | 14.9 | 47.3 | 158.8 | 148.5 | 165.3 | 156.6 |
| 15 | 9 | 3 | 1.838 | 3.299 | 3.150 | 8.286 | 9.7 | 17.0 | 16.2 | 42.8 | 190.1 | 194.0 | 194.9 | 193.5 |
| Additional treatment | | 1 | 1,731 | 3.700 | 3.483 | 8.914 | 9.7 | 18.5 | 18.7 | 46.8 | 179.1 | 200.0 | 186.6 | 190.3 |
| Additional treatment | | 2 | 1,510 | 3.793 | 4.838 | 10.141 | 8.4 | 16.4 | 23.3 | 48.0 | 180.6 | 231.8 | 207.9 | 211.3 |
| Additional treatment | | 3 | 2,482 | 2.921 | 3.582 | 8.986 | 13.7 | 15.0 | 17.8 | 46.5 | 181.6 | 194.7 | 200.9 | 193.2 |
