# Supplementary material for: Influence of drainage and nutrient-solution nitrogen and potassium concentrations on the agronomic behavior of bell-pepper plants cultivated in a substrate
Source: PLoS One. 2017 Jul 5;12(7):e0180529. doi: 10.1371/journal.pone.0180529 (PMC5498029; doi:10.1371/journal.pone.0180529)
Supplement: S4 Table — (DOCX) [file pone.0180529.s004.docx]

**S4 Table. Percentage of blossom-end rot for the early harvest (BEREH), intermediate harvest (BERIH), late harvest (BERLH) and total harvest (BERTH).**

| **N concentration** | **K concentration** | **Replicate** | **BEREH** | **BERIH** | **BERLH** | **BERTH** |
| --- | --- | --- | --- | --- | --- | --- |
| 6 | 3 | 1 | 0.0 | 0.0 | 0.0 | 0,0 |
| 6 | 3 | 2 | 0.0 | 0.0 | 0.0 | 0,0 |
| 6 | 3 | 3 | 1.8 | 0.0 | 0.0 | 0,4 |
| 6 | 5 | 1 | 17.9 | 0.0 | 1.1 | 2,8 |
| 6 | 5 | 2 | 0.0 | 0.0 | 0.0 | 0,0 |
| 6 | 5 | 3 | 3.6 | 0.0 | 0.0 | 0,9 |
| 6 | 7 | 1 | 0.0 | 0.0 | 0.0 | 0,0 |
| 6 | 7 | 2 | 0.0 | 0.0 | 0.0 | 0,0 |
| 6 | 7 | 3 | 0.0 | 0.0 | 0.0 | 0,0 |
| 6 | 9 | 1 | 0.0 | 0.0 | 0.0 | 0,0 |
| 6 | 9 | 2 | 0.0 | 1.1 | 0.0 | 0,4 |
| 6 | 9 | 3 | 0.0 | 0.0 | 0.0 | 0,0 |
| 9 | 3 | 1 | 1.7 | 2.1 | 0.0 | 1,2 |
| 9 | 3 | 2 | 0.0 | 0.0 | 0.0 | 0,0 |
| 9 | 3 | 3 | 5.4 | 1.1 | 0.0 | 1,7 |
| 9 | 5 | 1 | 1.4 | 1.9 | 0.9 | 1,4 |
| 9 | 5 | 2 | 0.0 | 1.3 | 1.4 | 1,0 |
| 9 | 5 | 3 | 0.0 | 0.0 | 0.0 | 0,0 |
| 9 | 7 | 1 | 2.0 | 1.8 | 1.4 | 1,7 |
| 9 | 7 | 2 | 0.0 | 0.0 | 0.0 | 0,0 |
| 9 | 7 | 3 | 2.4 | 1.1 | 1.2 | 1,5 |
| 9 | 9 | 1 | 0.0 | 0.0 | 7.3 | 2,8 |
| 9 | 9 | 2 | 5.3 | 0.0 | 9.0 | 4,8 |
| 9 | 9 | 3 | 1.5 | 0.0 | 0.0 | 0,4 |
| 12 | 3 | 1 | 23.8 | 10.0 | 0.0 | 11,3 |
| 12 | 3 | 2 | 9.5 | 9.3 | 4.3 | 7,6 |
| 12 | 3 | 3 | 0.0 | 2.1 | 0.0 | 0,7 |
| 12 | 5 | 1 | 1.4 | 15.7 | 10.6 | 10,6 |
| 12 | 5 | 2 | 61.4 | 21.4 | 11.5 | 26,1 |
| 12 | 5 | 3 | 3.0 | 4.7 | 1.1 | 3,0 |
| 12 | 7 | 1 | 1.3 | 1.0 | 0.0 | 0,8 |
| 12 | 7 | 2 | 2.8 | 4.9 | 6.3 | 4,8 |
| 12 | 7 | 3 | 0.0 | 1.1 | 5.4 | 2,5 |
| 12 | 9 | 1 | 1.3 | 5.7 | 2.7 | 3,5 |
| 12 | 9 | 2 | 5.0 | 8.2 | 11.6 | 8,7 |
| 12 | 9 | 3 | 3.1 | 9.7 | 1.0 | 5,0 |
| 15 | 3 | 1 | 11.7 | 13.8 | 11.7 | 12,7 |
| 15 | 3 | 2 | 16.5 | 16.4 | 3.7 | 12,9 |
| 15 | 3 | 3 | 15.6 | 11.5 | 3.3 | 9,8 |
| 15 | 5 | 1 | 17.3 | 16.1 | 0.0 | 12,6 |
| 15 | 5 | 2 | 24.5 | 25.8 | 19.2 | 23,7 |
| 15 | 5 | 3 | 2.5 | 2.0 | 0.9 | 1,7 |
| 15 | 7 | 1 | 8.3 | 22.8 | 0.0 | 13,0 |
| 15 | 7 | 2 | 11.0 | 18.0 | 9.1 | 13,6 |
| 15 | 7 | 3 | 11.3 | 18.4 | 3.3 | 12,3 |
| 15 | 9 | 1 | 14.1 | 19.5 | 4.4 | 13,4 |
| 15 | 9 | 2 | 11.3 | 15.1 | 26.1 | 18,0 |
| 15 | 9 | 3 | 6.5 | 3.8 | 0.0 | 3,0 |
| Additional treatment | | 1 | 0,3 | 0.3 | 0.0 | 0.7 |
| Additional treatment | | 2 | 0,0 | 0.0 | 0.0 | 0.0 |
| Additional treatment | | 3 | 0,0 | 0.3 | 0.0 | 0.3 |
